# Simultaneous Contact Sensing and Characterizing of Mechanical and Dynamic Heat Transfer Properties of Porous Polymeric Materials

**DOI:** 10.3390/ma10111249

**Published:** 2017-10-30

**Authors:** Bao-Guo Yao, Yun-Liang Peng, De-Pin Zhang

**Affiliations:** College of Mechatronics Engineering, China Jiliang University, Hangzhou 310018, China; 15757164756@163.com (Y.-L.P.); zhangdp1992@163.com (D.-P.Z.)

**Keywords:** mechanical properties, dynamic heat transfer, contact sensing, characterizing, measurement system, porous polymeric materials

## Abstract

Porous polymeric materials, such as textile fabrics, are elastic and widely used in our daily life for garment and household products. The mechanical and dynamic heat transfer properties of porous polymeric materials, which describe the sensations during the contact process between porous polymeric materials and parts of the human body, such as the hand, primarily influence comfort sensations and aesthetic qualities of clothing. A multi-sensory measurement system and a new method were proposed to simultaneously sense the contact and characterize the mechanical and dynamic heat transfer properties of porous polymeric materials, such as textile fabrics in one instrument, with consideration of the interactions between different aspects of contact feels. The multi-sensory measurement system was developed for simulating the dynamic contact and psychological judgment processes during human hand contact with porous polymeric materials, and measuring the surface smoothness, compression resilience, bending and twisting, and dynamic heat transfer signals simultaneously. The contact sensing principle and the evaluation methods were presented. Twelve typical sample materials with different structural parameters were measured. The results of the experiments and the interpretation of the test results were described. An analysis of the variance and a capacity study were investigated to determine the significance of differences among the test materials and to assess the gage repeatability and reproducibility. A correlation analysis was conducted by comparing the test results of this measurement system with the results of Kawabata Evaluation System (KES) in separate instruments. This multi-sensory measurement system provides a new method for simultaneous contact sensing and characterizing of mechanical and dynamic heat transfer properties of porous polymeric materials.

## 1. Introduction

Porous polymeric materials, such as textile fabrics, are widely used in our daily lives for garment and household products. When our body skin contacts porous polymeric materials, a series of sensations, such as roughness/smoothness and coolness/warmness occur, which greatly affects the wearing comfort.

When textile fabric is touched with the fingers of a human hand, many psychological sensations such as stiffness or rigidity, softness or hardness are perceived [1]. The handle properties of textile fabric, which describe the sensations during contact between textile fabric and the human hand, are related to wearing comfort and can be objectively tested by measuring the fundamental physical and mechanical sensory signals and simulating the dynamic contact and psychological judgment processes of the human hand.

The concept of fabric handle has long been used in the textile and clothing industries as a description of fabric quality and prospective performance [2]. It primarily influences the comfort sensations and aesthetic qualities of clothing, which may be the key factors for consumers in purchasing clothing and other textile products. Scientific research involving the testing and evaluation methods of fabric handle properties were conducted extensively and mainly focused on the judgment of thermal and mechanical properties, such as friction and bending properties [3,4,5,6,7,8,9,10,11,12,13,14,15,16,17].

The Kawabata Evaluation System (KES) was the first available and reliable solution for measuring the mechanical and heat transfer properties of textile fabric [3,4]. The KES system is composed of five instruments: the Compression Tester, Tensile and Shearing Tester, Surface Tester, Pure Bending Tester, and Thermolabo II. These five instruments were developed to simulate and test the cool-warm performance and the mechanical behavior of the textile fabrics during fabric-skin contact and human hand judgment processes. Based on the Kawabata KES system, Mitsuo studied fabric handle and its basic mechanical properties for various kinds of fabrics [6]. Gulrajani et al. assessed the low-stress mechanical properties of enzymatic-treated fabrics in the Kawabata system [8]. Chan et al. adopted the KES system to investigate the mechanical properties of uniform fabrics, so as to identify the design criteria for uniform clothing [11]. Varghese and Thilagavathi investigated the influence of fabric specification on total hand value, stretch properties, and pressure comfort. The effects of body shape and fabric mechanical properties on garment pressure were also analyzed [12]. Derler et al. proposed a realistic skin model using a force plate in combination with an objective friction test method, using different silicone and polyurethane materials as mechanical skin equivalents for assessing the surface roughness of the textiles [14].

The FAST (Fabric Assurance by Simple Testing) system is another set of apparatuses and test methods, developed by the CSIRO (Commonwealth Scientific and Industrial Research Organization) in Australia, which can measure the properties of bending, relaxation shrinkage and expansion, extensibility of the fabric in warp, weft and bias directions, and the thickness at two predetermined loads, on individual apparatuses [5]. The bending rigidity parameters of textile materials, defined at low-stress loads, were investigated by KES and FAST testers, respectively, to set the relationship between these two bending rigidity parameters [17].

However, these two measurement systems and solutions have to measure the thermal and mechanical performances separately on several instruments, and consequently the test time is long, and the cost is high. Furthermore, these two methods do not achieve the simultaneous perception of thermal and mechanical performance and therefore do not consider the interactions between different aspects of mechanical properties and dynamic heat transfer. Therefore, it is highly desirable to develop a test method for simultaneous sensing of the contact process and integration testing of mechanical and dynamic heat transfer properties of porous polymeric materials in one instrument.

In the literature, many studies have reported the measurement of mechanical and thermal properties of polymeric materials, including textile fabrics [18,19,20,21,22,23,24,25]. Darden and Schwartz proposed a framework to draw correlations between human sensory outcomes, such as smoothness and leather-like feel, and quantitative physical properties, such as friction coefficient and elastic modulus, for polymer fabrics [19]. Ullah et al. performed tensile tests to measure the mechanical properties of jute yarns, including tensile strength, modulus of elasticity, and strain to failure. This study had the advantage of quantifying the uncertainty associated with the mechanical properties, using both statistical analyses and possibility distributions [20]. Burg et al. presented a steady state measurement technique for sample thermal resistance, based on the heat flow meter bar approach, made up of two copper blocks and temperature measurements from thermocouples, which could be applied for the thermal conductivity measurement of polymer materials [21]. Acquarelli et al. produced a new sensor made of a vinyl-ester polymer composite filled with multilayer graphene nanoplatelets (MLG), through an innovative capillary rise method [22]. This method had the advantage of having an application in strain sensing and structural health monitoring by obtaining the electro-mechanical properties of the polymeric composite sensor. Claramunt et al. developed a process to produce high-performance cement-based composites, reinforced with flax nonwoven fabrics, and took advantage of flexural and direct tension tests for analyzing the influence of the fabric structure—thickness and entanglement—on mechanical behavior [23]. These test methods and instruments helped with the development of the integration measurement of mechanical and dynamic heat transfer properties of porous polymeric materials.

This paper reports the development of a new measurement system and method based on physical sensors and a virtual instrument for simultaneous sensing of the contact process and integrated measurement and characterization of mechanical and dynamic heat transfer properties of porous polymeric materials, such as textile fabrics in a single instrument. The multi-sensory measurement system and method can measure, record and analyze the dynamic heat transfer and mechanical sensory signals of porous polymeric materials simultaneously in one instrument during the simulated dynamic contact process. The initial ideas, the simple and immature device, and some initial trials have been reported in the prophase studies of this research [26].

## 2. Contact Sensing Principle and Evaluation Method

### 2.1. The Multi-Sensory Measurement System

A multi-sensory measurement system, based on physical sensors and a virtual instrument was developed, to simulate the dynamic contact process during human hand contact with porous polymeric materials, and simultaneously sense the mechanical and dynamic heat transfer signals with consideration of the interactions between different aspects of contact feels.

**Figure 1:**
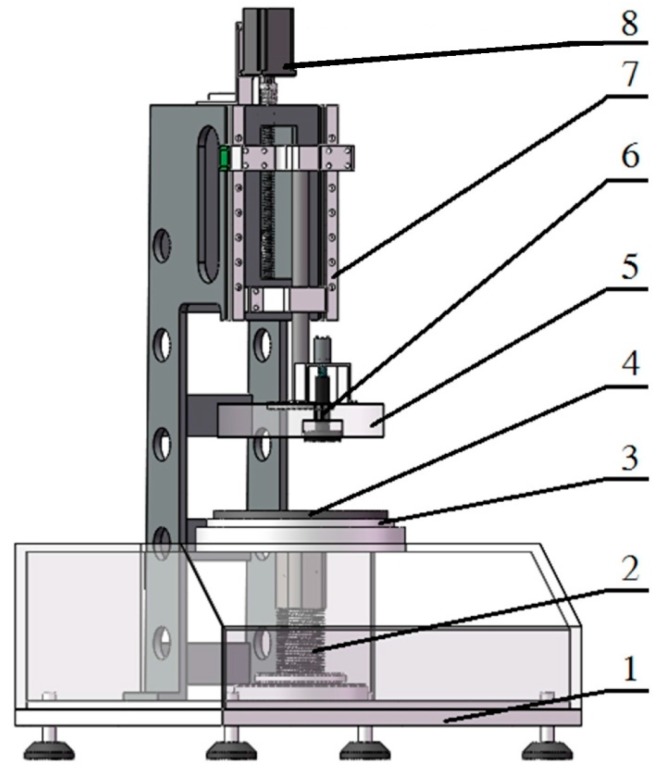
Mechanical structure of the multi-sensory measurement system.

The mechanical equipment of the multi-sensory measurement system consists of the following principal units, as shown in Figure 1: equipment base (1); spring (2); bending and twisting ring (3); lower measuring head (4); upper measuring head (5); smoothness measuring component (6); linear drive component (7); and head motion motor (8).

The top surface of the lower measuring head (4) is used as a platform for placing the test samples. The upper measuring head (5) can move up and down, which is operated by the linear drive component (7) and the head motion motor (8). The linear drive component contains the linear slide rail, slide table and ball screw. A bending and twisting ring (3) is set on the equipment base (1), which surrounds the lower measuring head. The smoothness measuring component (6) is integrated in the upper measuring head for the surface smoothness measurement of the sample. There is a spring (2) between the lower measuring head and the equipment base. Therefore, the lower measuring head can move down some distance when it is contacted by the upper measuring head; this ensures flexible contact and avoids damage.

Before testing, the upper measuring head is heated to the specified temperature. When the test starts, the upper measuring head moves downward and clamps the sample between the two surfaces of the upper measuring head and the lower measuring head. As a result of the applied pressure, the whole system of two measuring heads continues to move down, bending and twisting the sample just like grasping and kneading the sample using human hand and applying a certain amount of pressure on the pressure-sensing bending and twisting ring. In a few seconds, the lower measuring head reaches the lowest position according to the specified pressure (4.14 kPa, ASTM D1777) of the spring, and the smoothness measuring component starts to work towards obtaining the surface smoothness performance of the test sample. The two measuring heads remain fixed on the test sample for thirty seconds, and then the upper measuring head moves up automatically and returns to its original position.

During testing, the two measuring heads’ positions and sample thickness are recorded by two displacement sensors. The pressure on the pressure-sensing bending and twisting ring and the total pressure applied on the lower measuring head are measured by the contact force sensors and the pressure sensors to monitor the bending and twisting, and the compression changes during the measurement. Simultaneously, the heat flowing through the sample and the temperature of the two measuring heads are measured by the heat flux sensor and the temperature sensors. A strain gage installed in the smoothness measuring component is applied for the smoothness performance measurement.

The multi-sensory measurement system can be employed for simulating the dynamic contact and psychological judgment processes of human hand and integration testing and characterizing of the mechanical and dynamic heat transfer properties of porous polymeric materials by measuring surface smoothness, compression resilience, bending and twisting, and dynamic heat transfer signals simultaneously. It provides a relative and objective measurement method, in order to sense the contact process and differentiate between the mechanical and dynamic heat transfer properties of different samples.

#### 2.1.1. Surface Smoothness

**Figure 2:**
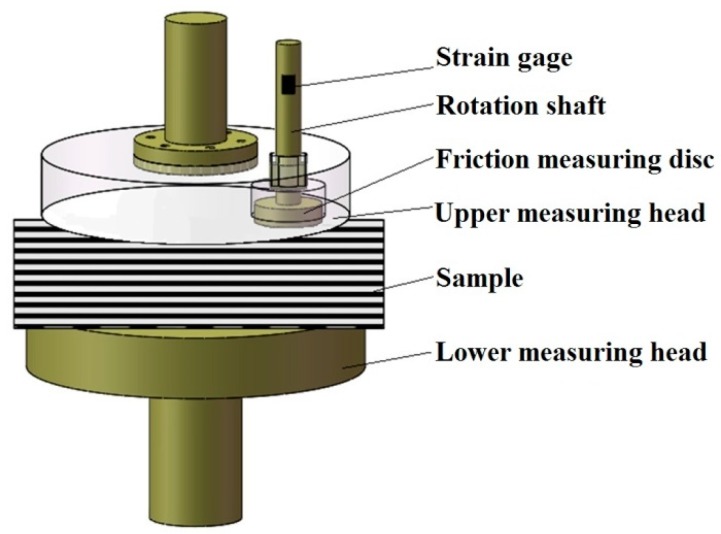
Surface smoothness measurement.

A diagram of the surface smoothness measurement is shown in Figure 2. The smoothness measuring component contains a friction measuring disc, rotation shaft and strain gage. When the upper and lower measuring heads reach the lowest position, a DC motor, installed on the upper measuring head, is switched on to drive the friction measuring disc by the rotation shaft to conduct a reciprocal rotation on the test sample surface. A strain gage, which is fixed on the rotation shaft, is used to dynamically measure the friction torque changes vs. time. The friction force can be calculated from the measured friction torque. Since the friction measuring disc floats on the rotation shaft in the vertical direction, the weight of the disc, G, is the normal pressure of the disc to the sample.

#### 2.1.2. Compression Resilience

In the measurement process, the test sample experiences two stages of compression and resilience, since the sample is elastic. The compression load is dynamically and automatically measured and recorded as the mean value of the three pressure sensors installed in the interlayer of the lower measuring head. The positions of the two measuring heads are measured by the displacement sensors, and the sample thickness thus can be calculated. Subsequently, the ratio of the compression load of the resilience stage to the compression stage is calculated, to evaluate the elastic and soft or stiff performances of the test sample.

#### 2.1.3. Bending and Twisting

**Figure 3:**
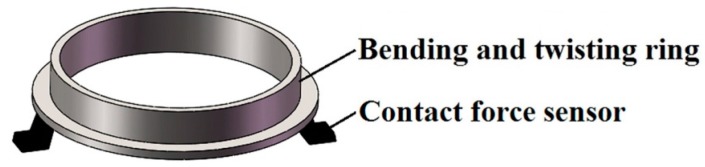
Bending and twisting ring.

**Figure 4:**
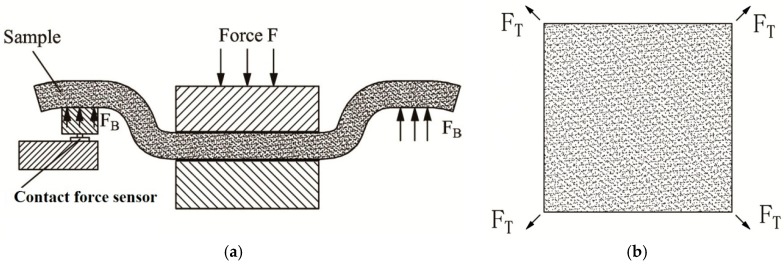
Bending and twisting performance measurement: (**a**) bending; (**b**) twisting.

The bending and twisting ring (Figure 3) is applied in the measurement of bending and twisting performances of the test sample. Four contact force sensors are evenly distributed on the bottom surface of the bending and twisting ring for the measurement of the overall characteristics of the bending (Figure 4a) and twisting (Figure 4b) properties. The bending and twisting load is dynamically measured and averaged by the four contact force sensors.

#### 2.1.4. Dynamic Heat Transfer

Before testing, the upper measuring head is heated to a 10 °C higher temperature than the lower measuring head, by a heating element implanted inside, which is the temperature difference between the hand surface and environment in warm conditions. When the two measuring heads clamp the test sample, the heat flows from the upper measuring head, through the sample, towards the lower measuring head, and the dynamic heat transfer can be monitored by a thin film heat flux sensor. The heat flux sensor is installed using conventional epoxies and placed in intimate contact with the surface of the lower measuring head. The thermal resistance temperature sensors are used to monitor the temperature changes of the two measuring heads during the measurement.

#### 2.1.5. Virtual Instrument System

In total, six different kinds of sensors, for measuring pressure, displacement, strain gage, contact force, heat flux and temperature, are used in this measurement system. All analog signals from the sensors during the measurement are processed and acquired by a virtual instrument (VI) system.

**Figure 5:**
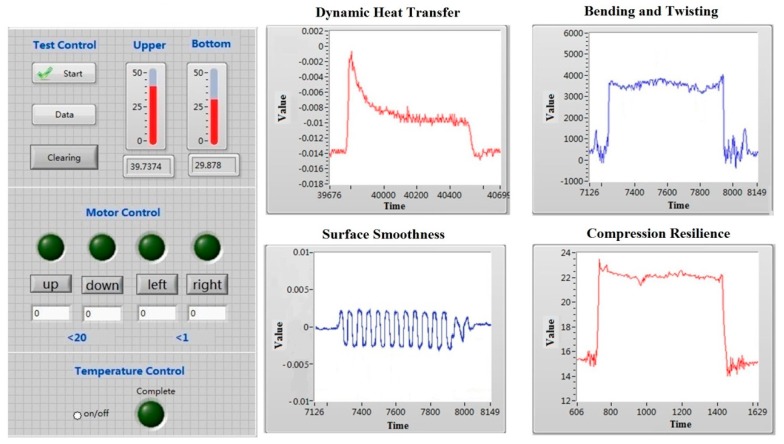
Front panel interface of the virtual instrument system.

The front panel interface of the VI system, including the solution of data acquisition and measurement control, is shown in Figure 5. The charts of bending and twisting, compression resilience, surface smoothness and dynamic heat transfer are respectively used for the display of dynamic changes of the measured signals. The bending and twisting chart shows the bending and twisting load curve, based on signals from the sensors of the pressure-sensing bending and twisting ring. The compression resilience chart illustrates the changes in compression load. The surface smoothness chart presents the measurement of the friction force from the strain gage. The dynamic heat transfer chart displays the heat flux curve.

### 2.2. Indices Definitions of Mechanical and Dynamic Heat Transfer Properties

Derived from the recorded data and the measured curves, a series of indices have been defined for the evaluation and characterization of the mechanical and dynamic heat transfer properties of porous polymeric materials.

The defined indices are classified, according to the homologous subjective psychological sensations, into four groups: the bending and twisting properties, the compression resilience properties, the surface smoothness properties and the dynamic heat transfer properties.

#### 2.2.1. The Bending and Twisting Properties

(1) MBTF: maximum bending and twisting load

The maximum bending and twisting load (MBTF) is defined as:

(1) $$\mathrm{MBTF}=\mathrm{BTF}\left(t\right){|}_{\max}$$

where *t* is the measurement time, and BTF(*t*) is the bending and twisting load vs. time.

(2) BTRD: bending and twisting rigidity descending

**Figure 6:**
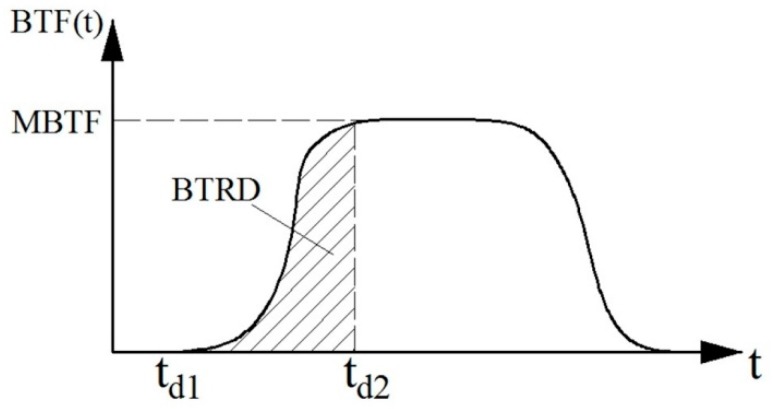
Typical curve of the bending and twisting load, MBTF: maximum bending and twisting load; BTRD: bending and twisting rigidity descending; BTF: bending and twisting load vs. time; *t_d_*_1_: time when the upper measuring head just touches the sample during the descending process; *t_d_*_2_: the time when the upper measuring head reaches the lowest position.

The typical bending and twisting load curve is shown in Figure 6. The BTRD (bending and twisting rigidity descending) is defined as the integral of bending and twisting load curve during the descending process of the upper measuring head.

(2) $$\mathrm{BTRD}=\int_{t_{d1}}^{t_{d2}}\mathrm{BTF}\left(t\right)dt$$

where *t_d_*_1_ is the time when the upper measuring head just touches the sample during the descending process and *t_d_*_2_ is the time when the upper measuring head reaches the lowest position.

#### 2.2.2. The Compression Resilience Properties

(1) CRD: compression rigidity descending

The integral of the compression load curve during the descending process of the upper measuring head is calculated as the compression rigidity descending (CRD).

(3) $$\mathrm{CRD}=\int_{t_{d1}}^{t_{d2}}\mathrm{CL}\left(t\right)dt$$

where CL(*t*) is the compression load vs. time.

(2) RR: resilience ratio

The integral of the compression load curve can be calculated as the compression rigidity, and the ratio of the compression rigidity of the resilience stage to the compression rigidity of compression stage is calculated to obtain the resilience ratio (RR).

(4) $$\mathrm{RR}=\frac{\int_{t_{a1}}^{t_{a2}}\mathrm{CL}\left(t\right)dt}{\mathrm{CRD}}$$

where *t_a_*_1_ is the time when the upper measuring head begins to ascend to the original position and *t_a_*_2_ is the time when the upper measuring head has just left the test sample.

#### 2.2.3. The Surface Smoothness Properties

(1) RSI: relative smoothness index

**Figure 7:**
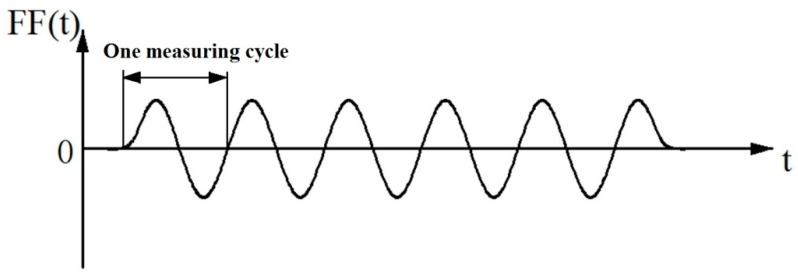
Typical curve of friction force, FF(*t*): friction force vs. time.

**Figure 8:**
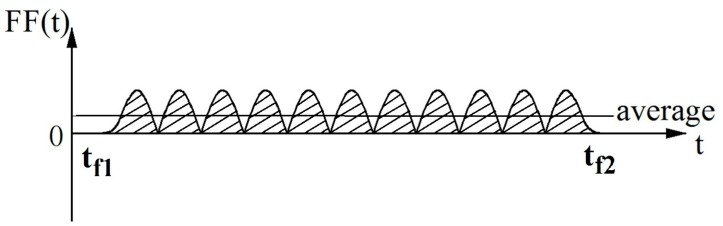
Friction force intensity, *t_f_*_1_: the time when the friction measuring disc starts rotating; *t_f2_*: the time when the friction measuring disc has just stopped rotating.

Figure 7 shows the typical friction force curve during the measurement. The relative smoothness index (RSI) is defined as the reciprocal of the dynamic friction coefficient for the surface smoothness properties. RSI describes the relative smoothness degree of the test sample.

(5) $$\mathrm{RSI}=\frac{1}{f_{d}}$$

where *f_d_* is the dynamic friction coefficient and can be calculated as the average value (Figure 8) of the friction forces, during the movement of the friction measuring disc, divided by the normal pressure, i.e., the weight of the friction measuring disc, G.

(6) $$f_{d}=\frac{\int_{t_{f1}}^{t_{f2}}\left|\mathrm{FF}\left(t\right)\right|dt}{G\left(t_{f2}-t_{f1}\right)}$$

where FF(*t*) is the friction force vs. time, *t_f_*_1_ is the time when the friction measuring disc starts rotating, and *t_f_*_2_ is the time when the friction measuring disc has just stopped rotating.

(2) FI: intensity of friction force

The area of the friction force curve and the time axis can be calculated as the friction force intensity (FI), as shown in Figure 8.

(7) $$\mathrm{FI}=\int^{\;}\left|\mathrm{FF}\left(t\right)\right|dt$$

#### 2.2.4. The Dynamic Heat Transfer Properties

(1) DHI: dynamic heat flow index

**Figure 9:**
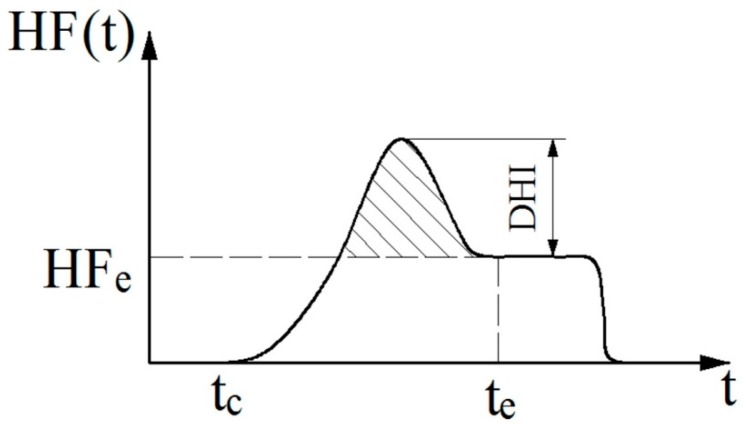
Typical curve of heat flow during the measurement process, HF(*t*): heat flux signal vs. time; HF_e_: average heat flux at equilibrium status; DHI: difference between dynamic heat flow ad heat transfer at equilibrium status; *t_c_*: time when the upper measuring head just contacts the sample and heat begins to transfer; *t_e_*: time when the heat transfer has just reached equilibrium status.

Figure 9 shows the typical heat flow curve during the measurement process. The maximum value of the heat flux during the measurement process, subtracted by the average heat flux at the equilibrium status of heat transfer is defined as the dynamic heat flow index (DHI).

(8) $$\mathrm{DHI}=\mathrm{HF}\left(t\right){|}_{\max}-{\mathrm{HF}}_{e}\;$$

where HF(*t*) is the heat flux signal vs. time, and HF_e_ is the average heat flux at equilibrium status. DHI represents the difference between dynamic heat flow and heat transfer at equilibrium status.

(2) TBA: thermal buffering ability

The thermal buffering ability (TBA) is defined as the average heat flow above the heat flux at equilibrium status during dynamic heat transfer.

(9) $$\{\begin{array}{c} \mathrm{TBA}=\frac{\int_{t_{c}}^{t_{e}}\left(\mathrm{HF}\left(t\right)-{\mathrm{HF}}_{e}\right)dt}{t_{e}-t_{c}}\\ \mathrm{HF}\left(t\right)\;=\;{\mathrm{HF}}_{e}\;\mathrm{if}\;\mathrm{HF}\left(t\right)<{\mathrm{HF}}_{e} \end{array},$$

where *t_c_* is the time when the upper measuring head just contacts the sample and heat begins to transfer, and *t_e_* is the time when the heat transfer reaches equilibrium status. TBA describes the thermal storage capability of the test sample during dynamic heat transfer.

All indices defined are summarized in Table 1.

## 3. Materials and Experiments

### 3.1. Experimental Setup

The test sample of porous polymeric material was cut to the size of 120 mm × 120 mm and any obvious wrinkles were removed by iron. The test sample was then placed in a conditioning room, which was controlled at 21 ± 1 °C and 65 ± 2% relative humidity (RH), in accordance with ASTM (American Society for Testing and Materials) D1776, for at least 24 h.

Five specimens of each test sample were prepared for the measurement. During testing, the specimen was placed on the lower measuring head in such a way that the specimen edges symmetrically crossed the diameter of the lower measuring head. The specimen edges, in this arrangement, overlapped the outer edge of the pressure-sensing bending and twisting ring surrounding the lower measuring head.

Before testing began, the temperature difference between the two measuring heads was controlled at 10 °C, in accordance with the general difference of temperatures between human hand skin and the environment in warm conditions.

### 3.2. Materials and Tests

**Figure 10:**
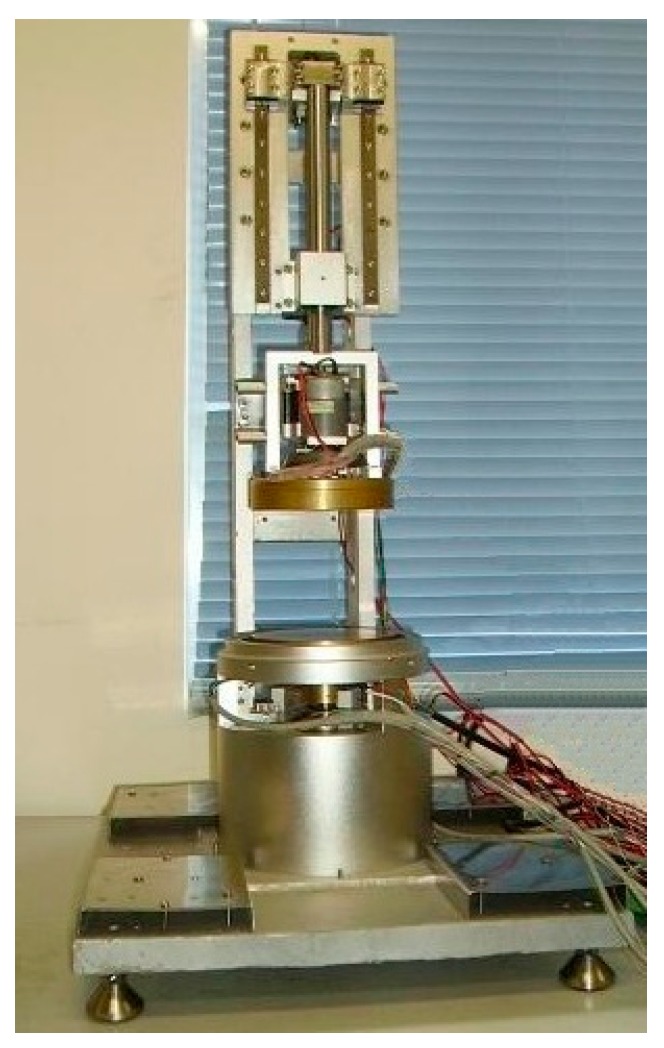
Mechanical equipment prototype of the measurement system.

Twelve types of samples of porous polymeric materials, which were purchased from department stores, were selected and tested in the experiments for mechanical and dynamic heat transfer performances. All the measurements were carried out in the conditioning room, where the environmental conditions were controlled, in accordance with the experimental protocol. For each set of sample, five pieces of specimens were prepared and measured. The experiments were conducted on an equipment prototype of the multi-sensory measurement system (Figure 10).

The basic structural parameters of the sample materials, such as construction, mass of unit area and thickness are listed in Table 2.

## 4. Results and Discussion

### 4.1. Objective Test Results

The test results of the evaluation indices by mean value and the standard deviation are summarized in Table 3.

A one-way ANOVA analysis for the evaluation indices was conducted to identify the significance of differences between mechanical and dynamic heat transfer performances of the test samples and the analysis results are summarized in Table 4.

The ANOVA table (Table 4) shows the influences of sample on individual measurement results to all indices. The results reveal that each evaluation index of mechanical and dynamic heat transfer properties was significantly different (*p* < 0.01) among different test samples. Therefore, the samples’ behaviors have significant influences on the mechanical and dynamic heat transfer properties of all evaluation indices.

**Figure 11:**
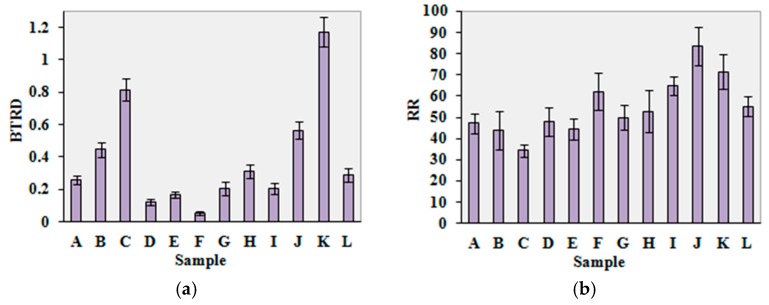

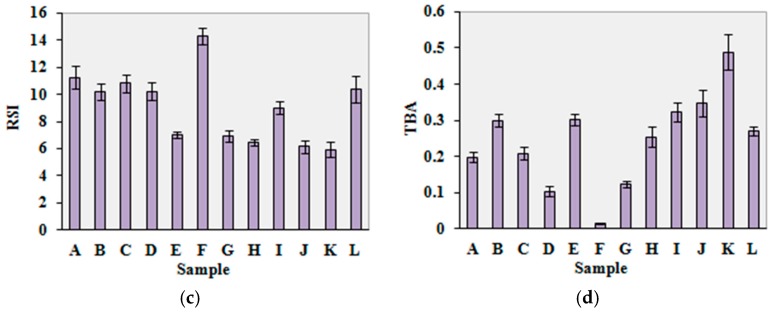
Bar charts of the test results, with bar errors for standard deviation: (**a**) bending and twisting rigidity descending (BTRD); (**b**) resilience ratio (RR); (**c**) relative smoothness index (RSI); (**d**) thermal buffering ability (TBA).

The bar charts of the test results for the evaluation indices, bending and twisting rigidity descending (BTRD), resilience ratio (RR), relative smoothness index (RSI) and thermal buffering ability (TBA) of the mechanical and dynamic heat transfer properties are shown in Figure 11. For the four groups of indices, each has one typical index for plotting.

The test results show that sample K was the stiffest sample, since it had the largest value of bending and twisting rigidity during the descending process of the upper measuring head. Sample J had the largest value of resilience ratio, indicating that sample J was the most elastic. Sample F was the smoothest surface property sample, since it had the largest value on the relative smoothness index. On the other hand, sample K had the roughest surface property with the smallest value on the relative smoothness index. Sample K had the largest thermal buffering ability value, and sample F had the smallest thermal buffering ability value. Therefore, the thermal buffering capability of sample K was the best, while the thermal buffering capability of sample F was the worst. Sample K was bleached denim. This kind of material is stiff and the surface is rough. Therefore, the bending and twisting rigidity was larger, and the surface smoothness value was smaller. Sample F was polyester material mesh fabric. The surface has various shapes of holes. This kind of material is light and thin in texture, and the touch feel is smooth and cool. Consequently, the surface smoothness value of sample F was larger and the thermal buffering capacity was poor. These are in accordance with the experimental results.

### 4.2. Correlation Analysis with Other Test Method

Since the KES system is widely used and accepted in this field, the test results of twelve typical samples by compression resilience and surface smoothness properties of the multi-sensory measurement system were compared with the test results of the Kawabata KES test method. The indices of other aspects of mechanical and dynamic heat transfer properties are dynamic indices or have no relative indices when compared with KES test method. The correlation analysis was conducted by comparing compression rigidity descending (CRD) and the relative smoothness index (RSI) of the multi-sensory measurement system in one instrument with the linearity of compression (LC) and mean friction coefficient (MIU) of the KES system in separate instruments. The correlations were significant (*p* = 0.00 < 0.05) for all the compared indices.

**Figure 12:**
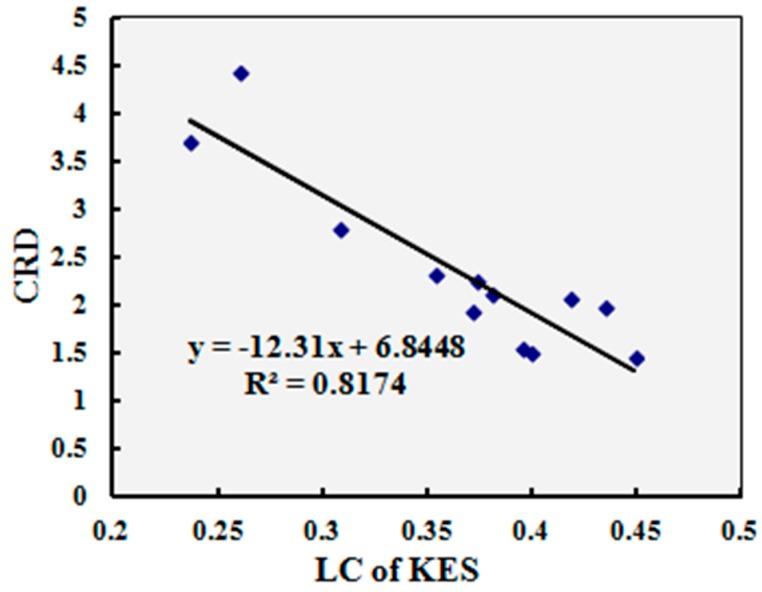
Relationship between compression rigidity descending (CRD) and LC of the Kawabata Evaluation System (KES) system, LC: linearity of compression.

**Figure 13:**
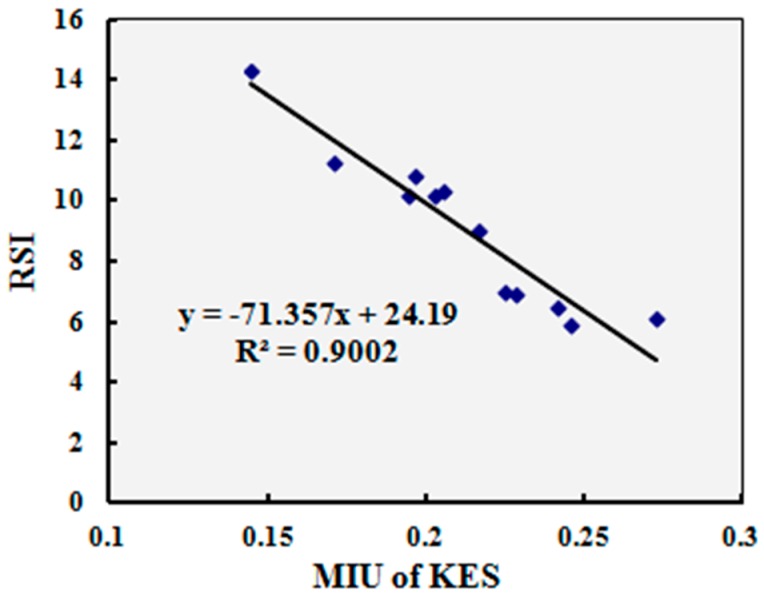
Relationship between relative smoothness index (RSI) and MIU of the KES system, MIU: mean friction coefficient.

Figure 12 and Figure 13 show the relationships between the CRD and RSI of the multi-sensory measurement system and LC and MIU of KES system. The correlation coefficients ($R^{2}$) were all greater than 0.8 for the compared indices, which indicates that the correlations are significant and the compared indices between the multi-sensory measurement system and the KES system have strong linear correlations.

The KES system measures the thermal and mechanical performance separately on several instruments, and consequently does not consider the interactions between different aspects of mechanical properties and dynamic heat transfer. The new multi-sensory measurement system can achieve the simultaneous contact sensing of mechanical and dynamic heat transfer properties of porous polymeric materials in one instrument, rather than using several separate instruments. Furthermore, the new multi-sensory measurement system can simulate dynamic contact and psychological judgment processes, during human hand contact with the porous polymeric materials, and has dynamic indices, such as bending and twisting rigidity descending (BTRD), intensity of friction force (FI), dynamic heat flow index (DHI) and thermal buffering ability (TBA) for characterizing the performance of the porous polymeric materials during the dynamic contact process.

### 4.3. Gage Capability, Repeatability and Reproducibility Study

In order to determine and assess the capability of the multi-sensory measurement system for mechanical and dynamic heat transfer properties, such as the two components of measurement error—the repeatability and reproducibility of the measurement system—capability, repeatability and reproducibility studies were carried out, based on the capability study experiment.

In the capability study experiment, another operator measured the same twelve samples, as shown in Table 2, using the same test conditions. Five specimens for each sample were also measured. The gage capability, repeatability and reproducibility were investigated based on the test results of two operators.

**Figure 14:**
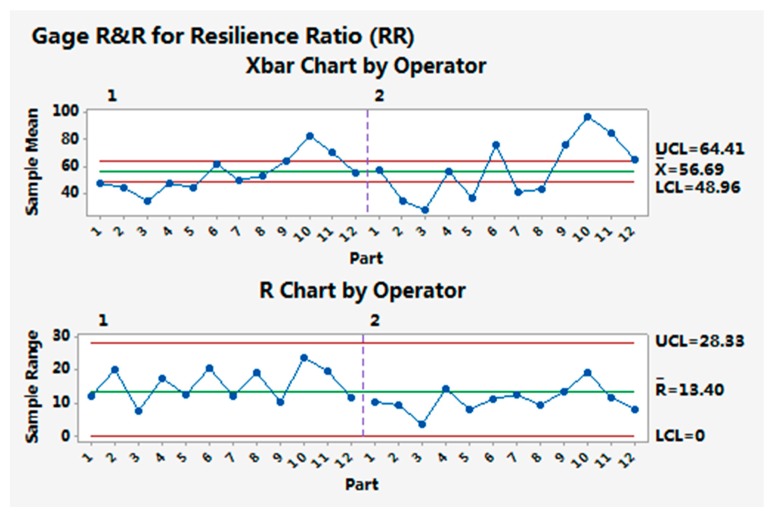
Gage capability result for the resilience ratio (RR).

**Figure 15:**
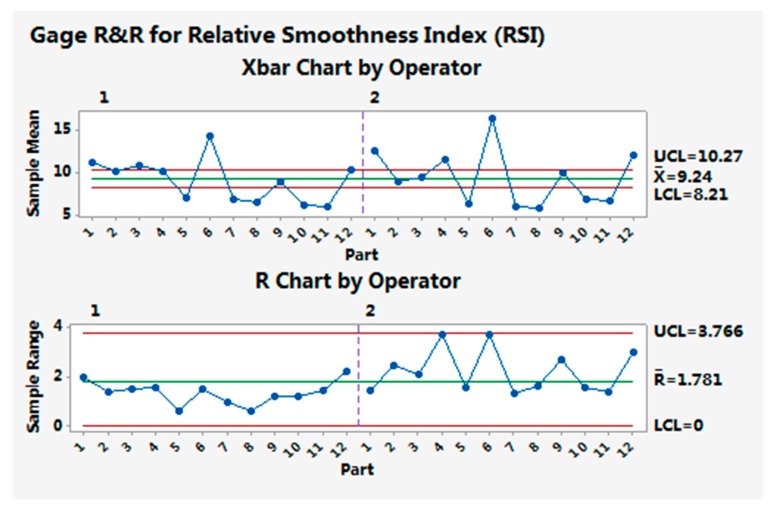
Gage capability result for the relative smoothness index (RSI).

Figure 14 and Figure 15 show the gage capability results for the resilience ratio and relative smoothness index, as examples of the analysis results for the evaluation indices.

The multi-sensory measurement system can distinguish the resilience ratios and relative smoothness indices between different samples, since the Xbar chart has out-of-control points. The R chart is in control, showing that the operator has no difficulty in making consistent measurements for the resilience ratio and the relative smoothness index. For other indices, similar results for the measurement system gage capability were obtained.

The investigation results of gage repeatability and reproducibility are summarized as Table 5. The values of gage repeatability, reproducibility and part-to-part are presented as the contribution percentages of variance components. The part-to-part means the contribution percentages caused by different samples.

The gage repeatability of all indices was less than 10%, indicating that the multi-sensory measurement system is able to make repeat measurements on the test samples with system variations below 10%. The gage reproducibility was also less than 10% for most indices, except RR (13.91%) and FI (11.19%), showing that different operators have no difficulty in making measurements of similar samples with small variations.

## 5. Conclusions

A multi-sensory measurement system and test method, based on physical sensors and a virtual instrument (VI) was developed to simulate the dynamic contact and psychological judgment processes of the human hand and objectively measure and characterize the mechanical and dynamic heat transfer properties of porous polymeric materials, such as textile fabrics, in one instrument, by simultaneously measuring and analyzing the surface smoothness, compression resilience, bending and twisting, and dynamic heat transfer signals that were acquired during a simulated dynamic contact process. Derived from the test data, a series of indices were defined, to characterize and evaluate the mechanical and dynamic heat transfer performance of the test samples.

Twelve types of samples of porous polymeric materials with different structural feature were tested. The results show that there were significant differences in the mechanical and dynamic heat transfer performances—including bending and twisting properties, compression resilience properties, surface smoothness properties and dynamic heat transfer properties—among the measured samples, for all defined indices.

The correlations were significant, when comparing the compression rigidity descending (CRD) and relative smoothness index (RSI) of the multi-sensory measurement system, in one instrument, with the linearity of compression (LC) and mean friction coefficient (MIU) of the KES system in separate instruments.

The capability study result for the gage repeatability and reproducibility revealed that the new measurement system is able to make repeat measurements on the test samples and different operators have no difficulty in making the measurements of similar samples with variations below 10%.

The multi-sensory measurement system provides a new method for simultaneous sensing of the contact process and integrated measurement and characterization of mechanical and dynamic heat transfer properties of porous polymeric materials.

## Figures and Tables

**Table 1:** Indices defined for mechanical and dynamic heat transfer properties.

Index	Definition	Unit
MBTF	maximum bending and twisting load	N
BTRD	bending and twisting rigidity descending	N·s
CRD	compression rigidity descending	N·s
RR	resilience ratio	
RSI	relative smoothness index	
FI	intensity of friction force	N·s
DHI	dynamic heat flow index	kW/m^2^
TBA	thermal buffering ability	kW/m^2^

**Table 2:** Sample structural parameters.

Sample Code	Sample Construction	Mass/Unit Area (g/m^2^)	Thickness (mm) at 4.14 kPa (ASTM D1777)
A	100% polyester, fleece	252	1.39
B	70% polyester 30% rayon	258	1.82
C	100% polyester	295	3.05
D	100% cotton	208	0.97
E	80% cotton 20% polyester, velour	279	1.38
F	100% polyester, mesh	97	0.33
G	100% cotton, twill	291	0.59
H	100% cotton, corduroy	307	0.91
I	100% polyester, fancy	243	0.56
J	100% cotton, denim	361	0.78
K	100% cotton, bleached	359	0.86
L	100% cotton, velvet	267	0.97

**Table 3:** Test results by mean values and standard deviation of the evaluation indices.

Sample	MBTF (N)	BTRD (N·s)	CRD (N·s)	RR	FI (N·s)	RSI	DHI (kW/m^2^)	TBA (kW/m^2^)
A	0.264 ± 0.012	0.258 ± 0.025	2.083 ± 0.317	47.329 ± 4.685	0.312 ± 0.025	11.265 ± 0.833	0.499 ± 0.037	0.198 ± 0.014
B	0.259 ± 0.005	0.446 ± 0.043	3.708 ± 0.459	44.159 ± 9.014	0.343 ± 0.016	10.209 ± 0.573	0.688 ± 0.056	0.299 ± 0.018
C	0.411 ± 0.016	0.817 ± 0.071	4.428 ± 0.455	34.537 ± 2.908	0.324 ± 0.019	10.831 ± 0.658	0.405 ± 0.060	0.209 ± 0.018
D	0.147 ± 0.005	0.122 ± 0.019	2.342 ± 0.401	48.198 ± 6.716	0.343 ± 0.024	10.242 ± 0.688	0.287 ± 0.015	0.104 ± 0.013
E	0.13 ± 0.017	0.171 ± 0.020	2.804 ± 0.299	44.351 ± 4.981	0.499 ± 0.018	7.02 ± 0.250	0.849 ± 0.038	0.302 ± 0.014
F	0.072 ± 0.002	0.054 ± 0.011	1.937 ± 0.223	62.138 ± 8.878	0.245 ± 0.010	14.305 ± 0.609	0.060 ± 0.005	0.014 ± 0.002
G	0.418 ± 0.011	0.206 ± 0.042	1.989 ± 0.150	50.03 ± 5.907	0.507 ± 0.032	6.928 ± 0.439	0.284 ± 0.020	0.124 ± 0.010
H	0.442 ± 0.008	0.31 ± 0.043	2.26 ± 0.192	52.911 ± 9.944	0.54 ± 0.022	6.493 ± 0.247	0.684 ± 0.039	0.254 ± 0.028
I	0.283 ± 0.008	0.207 ± 0.034	1.463 ± 0.216	64.944 ± 4.465	0.389 ± 0.021	9.028 ± 0.457	0.923 ± 0.053	0.322 ± 0.027
J	0.812 ± 0.016	0.567 ± 0.055	1.517 ± 0.137	83.769 ± 9.108	0.57 ± 0.047	6.165 ± 0.464	0.887 ± 0.037	0.348 ± 0.036
K	0.92 ± 0.015	1.174 ± 0.092	1.57 ± 0.167	71.555 ± 8.061	0.596 ± 0.060	5.92 ± 0.548	1.251 ± 0.074	0.489 ± 0.048
L	0.315 ± 0.011	0.289 ± 0.042	2.121 ± 0.262	55.14 ± 4.570	0.339 ± 0.032	10.383 ± 0.989	0.714 ± 0.041	0.271 ± 0.013

**Table 4:** One-way ANOVA analysis results of the evaluation indices.

Dependent Variable	Sum of Squares	df	Mean Square	F	*p* (sig.)
MBTF	3.686	11	0.335	2566.301	0.000
BTRD	5.826	11	0.530	243.180	0.000
CRD	44.444	11	4.040	46.652	0.000
RR	10,176.558	11	925.142	19.046	0.000
FI	0.762	11	0.069	75.462	0.000
RSI	367.567	11	33.415	92.832	0.000
DHI	6.119	11	0.556	290.096	0.000
TBA	0.869	11	0.079	145.623	0.000

**Table 5:** Gage repeatability and reproducibility results.

Indices	Repeatability (%)	Reproducibility (%)	Part-to-Part (%)
MBTF	0.33	0.45	99.22
BTRD	1.70	2.02	96.28
CRD	5.34	3.50	91.16
RR	9.29	13.91	76.80
FI	7.21	11.19	81.60
RSI	6.96	6.78	86.26
DHI	1.73	2.37	95.90
TBA	2.94	3.17	93.89
